# An InGaN/GaN Superlattice to Enhance the Performance of Green LEDs: Exploring the Role of V-Pits

**DOI:** 10.3390/nano8070450

**Published:** 2018-06-21

**Authors:** Mengling Liu, Jie Zhao, Shengjun Zhou, Yilin Gao, Jinfeng Hu, Xingtong Liu, Xinghuo Ding

**Affiliations:** 1 Key Laboratory of Hydraulic Machinery Transients (Wuhan University), Ministry of Education, Wuhan 430072, China; lml0305@whu.edu.cn (M.L.); zjie1994@whu.edu.cn (J.Z.); gyl1016@whu.edu.cn (Y.G.); 2017282080101@whu.edu.cn (J.H.); 2016202080010@whu.edu.cn (X.L.); xinghuo@whu.edu.cn (X.D.); 2 Center for Photonic and Semiconductor, School of Power and Mechanical Engineering, Wuhan University, Wuhan 430072, China

**Keywords:** green LEDs, InGaN/GaN superlattice, V-pits, external quantum efficiency

## Abstract

Despite the fact that an InGaN/GaN superlattice (SL) is useful for enhancing the performance of a GaN-based light-emitting diode (LED), its role in improving the efficiency of green LEDs remains an open question. Here, we investigate the influence of a V-pits-embedded InGaN/GaN SL on optical and electrical properties of GaN-based green LEDs. We recorded a sequence of light emission properties of InGaN/GaN multiple quantum wells (MQWs) grown on a 0- and 24-pair InGaN/GaN SL by using scanning electron microscopy (SEM) in combination with a room temperature cathodoluminescence (CL) measurement, which demonstrated the presence of a potential barrier formed by the V-pits around threading dislocations (TDs). We find that an increase in V-pit diameter would lead to the increase of V-pit potential barrier height. Our experimental data suggest that a V-pits-embedded, 24-pair InGaN/GaN SL can effectively suppress the lateral diffusion of carriers into non-recombination centers. As a result, the external quantum efficiency (EQE) of green LEDs is improved by 29.6% at an injection current of 20 mA after implementing the V-pits-embedded InGaN/GaN SL layer. In addition, a lower reverse leakage current was achieved with larger V-pits.

## 1. Introduction

GaN-based light-emitting diodes (LEDs) have been applied in many commercial areas, such as backlights for liquid crystal displays (LCDs), solid-state lighting, visible light communications, head-up displays, and optogenetics [1,2,3,4,5,6,7]. Generally, the white light emission is obtained by using a combination of phosphors with a blue LED. However, in this scheme, the light emission efficiency of a phosphor-converted white LED is limited due to the energy loss when higher energy photons are converted to lower energy photons. To solve this problem, the direct color mixing of red, green, and blue LEDs are generally considered as a sufficiently efficient method to eliminate optical conversion using phosphors. Nevertheless, it is difficult to obtain high-efficiency LEDs with an emission wavelength ranging from 500 nm to 600 nm by using GaN-based or AlGaInP-based materials, which is known as the “green gap” [8,9,10].

It has been reported that the “green gap” is caused by high-density threading dislocations (TDs), which result from a large mismatch in the lattice parameter between the GaN and the high-In-content InGaN. The TDs can severely reduce the quantum efficiency of green LEDs [11,12,13,14]. Additionally, highly strained InGaN/GaN green multiple quantum wells (MQWs), due to a large lattice mismatch between the high-In-content InGaN and the GaN, exhibit strong piezoelectric-field-induced quantum-confined stark effects (QCSE), resulting in a spatial separation of the wave functions of electrons and holes in the quantum well [15,16]. In the past few decades, much scientific effort has focused on approaches to improve the optical and electrical properties of green LEDs [17,18,19]. It was found that hexagonal V-pits having inverted pyramids with (10-11) faceted sidewalls were generally formed in InGaN/GaN MQWs [20,21,22,23]. Previous studies have demonstrated that the V-pits could impact the performance of LEDs, such as the leakage current, electrostatic discharge capabilities, and the radiative recombination efficiency [24,25,26,27]. An underlying InGaN/GaN superlattice (SL) embedded between an n-GaN and an MQW has been used to relax misfit strain [28,29,30,31]. However, the increasing SL pairs would accumulate strain energy and the partial strain relaxation would trigger the formation of V-pits [32]. The influence of V-pits and their energy barrier originating from (10-11) facets on optical and electrical properties of LEDs have been investigated [33,34]. It was observed that the presence of V-pits could efficiently suppress the lateral diffusion of excited carriers into non-radiative recombination centers at TDs [35]. Despite the fact that the insertion of an underlying InGaN/GaN SL before the growth of InGaN/GaN MQWs is known to enhance the device performance of LEDs, its role in improving the efficiency of green LEDs remains an open question.

In this work, we investigated the effect of a V-pits-embedded, 24-pair InGaN/GaN SL on the optical and electrical properties of green LEDs. Atomic force microscopy (AFM), scanning electron microscopy (SEM), and cathodoluminescence (CL) measurements were used to characterize the surface morphology and optical property of a green InGaN/GaN MQW. A detailed analysis of a V-pit’s structure was performed by using high-angle dark-field (HAADF) scanning transmission electron microscopy (STEM). Additionally, the potential barrier height around the V-pit was also investigated by CL. We found that V-pits play an important role in improving the external quantum efficiency (EQE) and reducing the reverse leakage current of green LEDs.

## 2. Materials and Methods

### 2.1. Growth and Device Fabrication

**Figure 1:**
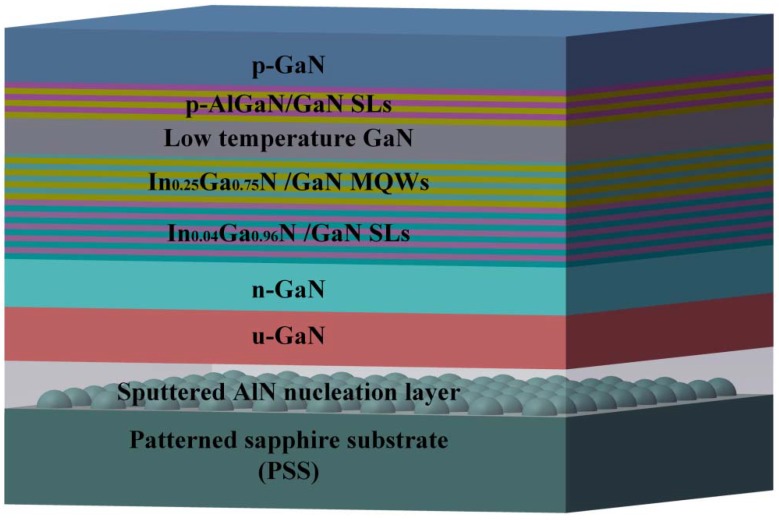
Schematic illustration of green light-emitting diode (LED) epitaxial structure. MQW, multiple quantum well; SL, superlattice.

The green MQW and LED samples used in this study were grown on a *c*-plane-patterned sapphire substrate by metal organic chemical vapor deposition (MOCVD). Trimethylindium (TMIn), trimethylgallium (TMGa), and ammonia (NH_3_) were used as gallium (Ga), indium (In), and nitrogen (N) sources, respectively. Silane (SiH_4_) and biscyclopentadienylmagnesium (CP_2_Mg) were used as the n-dopant and p-dopant source. Hydrogen was used as the carrier gas for the growth of the GaN layer while nitrogen was used as the carrier gas for the growth of the InGaN layer. The epitaxial structure of the green LEDs is composed of a 25-nm-thick sputtered AlN nucleation layer, a 2-μm-thick undoped GaN buffer layer, a 2.5-μm-thick Si-doped n-GaN layer, a 24-pair In_0.04_Ga_0.96_N (3 nm)/GaN (3 nm) SL at 800 °C, a 12-pair In_0.25_Ga_0.75_N (3 nm)/GaN (12 nm) MQW, a 60-nm-thick low temperature p-GaN layer, a 60-nm-thick p-AlGaN/GaN SL electron blocking layer, and a 285-nm-thick p-GaN capping layer. Figure 1 shows schematic illustration of the green LED epitaxial structure. To investigate the effect of the InGaN/GaN SL on optical and electrical properties of green LEDs, the 24-pair In_0.04_Ga_0.96_N (3 nm)/GaN (3 nm) was sandwiched between the In_0.25_Ga_0.75_N/GaN MQW active layer and the n-GaN layer. A green LED without an InGaN/GaN SL was also grown for reference. Additionally, the green MQWs without and with an InGaN/GaN SL were prepared for investigation of the surface morphology.

To form the mesa structure of the green LEDs and expose the n-GaN layer for the deposition of an n-type electrode, a standard photolithography process and BCl_3_-based inductively coupled plasma etching were performed. Next, an indium tin oxide (ITO) transparent conductive layer was deposited onto the p-GaN layer for current spreading followed by thermal annealing at 500 °C for 30 min in an N_2_ atmosphere. Finally, Cr/Au (30/300 nm) layers were evaporated onto the ITO layers and n-GaN layer for formation of the n-type and the p-type electrodes using an e-beam evaporator. Finally, the epitaxial green LED wafers were diced into chips with a size of 305 × 330 μm^2^.

### 2.2. Material Characterization and Measurement

AFM (Bruker, Karlsruhe, Germany), performed on a Bruker Multimode 8 in tapping mode, was used to determine the V-pit densities of green MQW samples. The surface morphology of green MQW samples was characterized by using an FEI Nova 3D (FEI, Hillsboro, OR, USA). CL measurements performed on an FEI Quanta 200F field emission SEM (FEI, Hillsboro, OR, USA) fitted with a Gatan Mono CL3+ (Gatan, Pleasanton, CA, USA) system under a vacuum of 10^−6^ Torr were used to further analyze the properties of V-pits. Cross-sectional and plan-view TEM samples were prepared by focus ion beam (FIB) milling using Ga ions at 30 kV, and then TEM images were taken with an FEI Talos F200X system at 200 kV (FEI, Hillsboro, OR, USA). X-ray diffraction (XRD) performed on a BEDE D1 (BEDE, Durham, UK) was used to characterize the crystalline quality of the green LEDs. Temperature-dependent photoluminescence (PL) experiments were conducted at temperatures from 5 K to 300 K using a He–Cd laser (λ = 325 nm) as the excitation source. The detection part of the PL system consisted of a charge-coupled device detector (Princeton Instruments PIX IS256, Trenton, NJ, USA) connected to a spectrometer (Princeton Instruments SP2500i, Trenton, NJ, USA). The light output power versus current and the current versus voltage characteristics of the green LEDs were measured by using a probe station system (NationStar, Foshan, China) [36].

## 3. Results and Discussion

**Figure 2:**
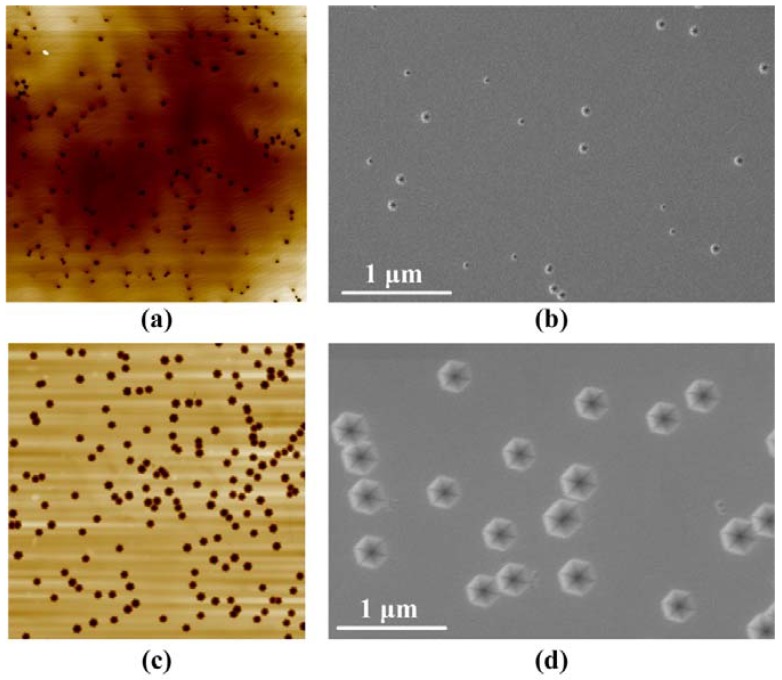
The 10 μm × 10 μm AFM and morphological SEM images of samples. (**a**,**b**) without an InGaN/GaN SL; (**c**,**d**) with an InGaN/GaN SL.

Figure 2 shows AFM and morphological SEM images of green MQW samples without and with an InGaN/GaN SL. The V-pit densities of the sample without and with an InGaN/GaN SL were 1.54 × 10^8^ and 1.75 × 10^8^ cm^−2^, respectively. A previous study revealed that stacking mismatch boundaries induced by stacking faults would trigger the formation of V-pits due to the strain relaxation [21]. As a result, a higher V-pits density was observed in the green MQW grown on an InGaN/GaN SL because of the accumulated strain energy. Additionally, the V-pit diameter of the MQW sample without and with an InGaN/GaN SL was determined to be about 99 and 280 nm, respectively, as shown in Figure 2c,d. Accordingly, the corresponding ratios of the V-pit area were 0.02 and 0.12 with a V-pit diameter of 99 and 280 nm, respectively.

**Figure 3:**
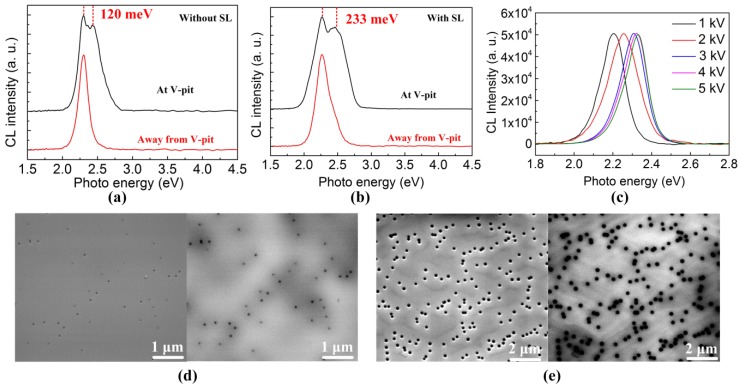
CL spectra measured from a green InGaN/GaN MQW (**a**) without an InGaN/GaN SL; and (**b**) with an InGaN/GaN SL at an acceleration voltage of 2 kV; (**c**) Depth-resolved CL spectra of the green InGaN/GaN MQW measured at various acceleration voltages. The SEM and CL images taken from the same area of the samples (**d**) without an InGaN/GaN SL; (**e**) with an InGaN/GaN SL.

To further analyze the optical properties of V-pits, the CL spectra of the green MQWs were measured around the V-pits and at a point away from the V-pits at an acceleration voltage of 2 kV at room temperature. As shown in Figure 3a,b, the CL spectra exhibited two emission components, which comprised a main energy peak identical to that taken from the point away from the V-pits and a higher-energy component corresponding to the emission energy of the sidewall MQW of the V-pits. In the case of the CL spectra for the green MQWs without an InGaN/GaN SL, the main energy is about 2.30 eV, and the higher-energy component is approximately 2.42 eV. The energy gap between the main energy and the higher-energy component was 120 meV, which indicated that a potential barrier was formed by the V-pit. However, in the case of the InGaN/GaN SL, the energy gap between the main energy and the higher-energy component was about 233 meV as shown in Figure 3b. The potential barrier height of the green MQW with an InGaN/GaN SL was 113 meV higher than that of the green MQW without an InGaN/GaN SL. The extended potential barrier height was attributed to the larger V-pit diameter. A CL intensity measurement with various acceleration voltages was also performed to obtain depth-resolved information on the optical property of the InGaN layer. Figure 3c shows the peak shift of the InGaN emission toward higher energy with increasing acceleration voltage from the outer to the inner region of the InGaN layer. The penetration depth of an electron is determined by the acceleration voltage of the electron beam during the CL measurement. It was previously reported that strain hinders the incorporation of In atoms in the InGaN lattice and is the driving force for the compositional pulling effect in InGaN films [37,38]. As the layer thickness increases, the InGaN film partially relaxes allowing more In atoms to be incorporated in the lattice. For electron energy from 1 kV to 5 kV, the region of maximum energy deposition progressively moves from the near-surface region to the deeper InGaN/GaN QW. The observed blue-shifts of the CL peak position with the increasing acceleration voltage from 1 kV to 5 kV can be explained by a decrease of In content over the penetration depth due to a compositional pulling effect. At an acceleration voltage of 5 kV, the penetration depth of an electron is estimated to be 125 nm. The thickness of the In_0.25_Ga_0.75_N/GaN green MQW (180 nm) is much larger than the depth of the electron penetration depth at 5 kV. Depth-resolved CL spectra of a green InGaN/GaN MQW measured at various acceleration voltages can exclude the possibility of part of the CL arising from the InGaN/GaN SL, which might result in a blue shift not related to the potential barrier around the dislocation.

As shown in Figure 3d,e, the CL intensity distribution images of the green LEDs without and with an InGaN/GaN SL along with SEM images were taken from the same area of the samples. It was obviously found that the V-pits, directly observable on the SEM images, coincide with dark spots in the CL images corresponding to the non-radiative recombination areas where the TD is located [39]. Generally, the excited charge carriers in the InGaN quantum wells are prone to diffuse laterally until they recombine radiatively or non-radiatively. The excited charge carriers can excite over the small V-pits with a relatively low potential barrier height and non-radiatively recombine at TDs. Therefore, it was observed in Figure 3d,e that the lateral size of the dark spot around small V-pits was larger than its physical size, suggesting that the non-radiative recombination regions are extended outside the V-pits. However, the presence of larger V-pits with a higher potential barrier could effectively suppress the lateral diffusion of the carriers into TDs, minimizing the size of the dark spot.

**Figure 4:**
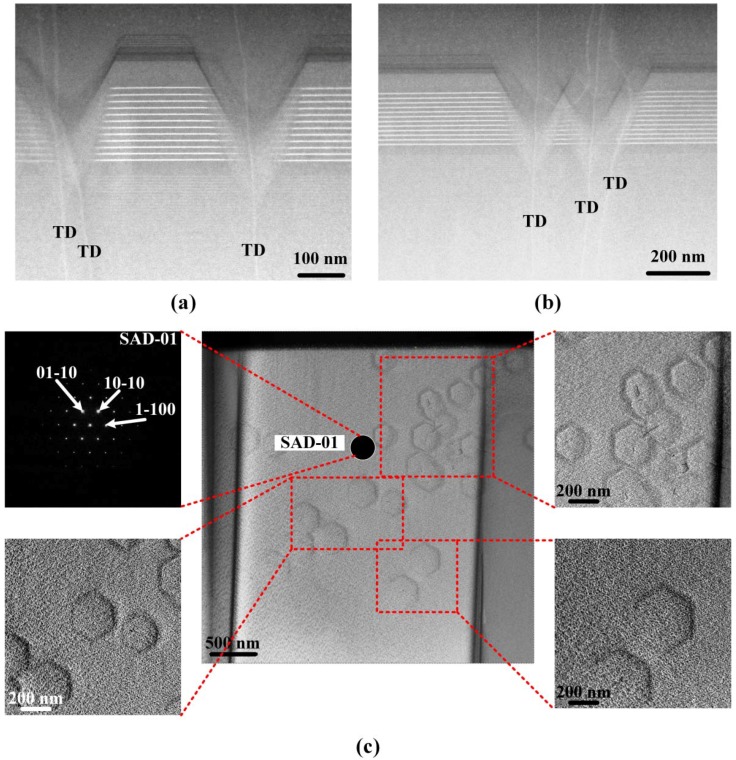
(**a**,**b**) The HAADF-STEM images showing a representative cross-section of V-pits; (**c**) Plan-view TEM images of a green LED specimen including layers of low temperature p-GaN, InGaN/GaN MQW, and a portion of n-GaN with the selected area diffraction (SAD) pattern of SAD-01.

Figure 4a,b show the cross-sectional HAADF-STEM images of a green In_0.25_Ga_0.75_N/GaN MQW grown on an In_0.04_Ga_0.96_N/GaN SL, where the V-pits connecting with TDs extend from the underlying SL and pass through the MQW to the low temperature p-GaN and p-AlGaN electron blocking layer. We found that large V-pits start to form at the last few SL pairs while small V-pits form at the first few quantum well pairs. Besides this, it was interesting to observe in Figure 4b that two adjacent V-pits merged and formed a wider W-defect section. A plan-view TEM analysis was performed to confirm the distribution of V-pits on the epilayer by bright-field imaging in the vicinity of the (0001) zone axis. Figure 4c shows the plan-view TEM images of a portion of a green LED epitaxial layer, including the low temperature p-GaN layer, the InGaN/GaN MQW, and a portion of the n-GaN layer along the (0001)-growth direction. The plan-view TEM images associated with selected area diffraction (SAD) patterns confirm that the V-pits were projected along (0001), and each side of the hexagon corresponds to intersections of the (0001) and (10-11) planes.

**Figure 5:**
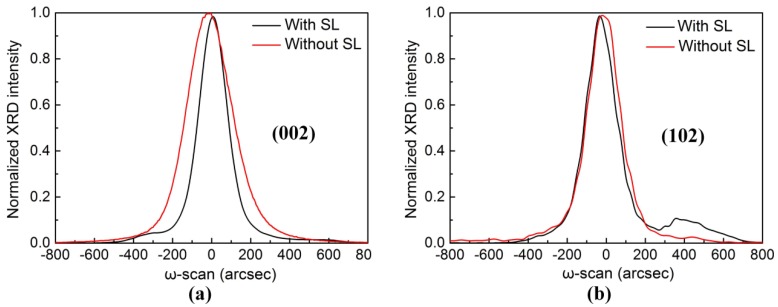
(**a**) Symmetric (002) and (**b**) asymmetric (102) XRD ω-scan rocking curves of green LEDs without and with an InGaN/GaN SL.

To elucidate the impact of the underlying InGaN/GaN SL on the crystalline quality of the green LEDs, we performed an X-ray diffraction (XRD) measurement on the green LEDs. Figure 5 shows symmetric (002) and asymmetric (102) ω-scan rocking curves of green LEDs without and with an InGaN/GaN SL. The full widths at half maximum (FWHMs) of the symmetric (002) rocking curves of green LEDs without and with an InGaN/GaN SL were about 265 and 165.5 arcsec, respectively. The FWHMs of the symmetric (102) rocking curve of green LEDs without and with an InGaN/GaN SL were about 194.2 and 181.5 arcsec, respectively. The FWHMs of symmetric (102) and (002) rocking curves of green LEDs with an InGaN/GaN SL are much smaller than those of green LEDs without an InGaN/GaN SL. We can reasonably conclude that the green LED with an InGaN/GaN SL has superior crystallite quality as compared with the green LED without an InGaN/GaN SL.

**Figure 6:**
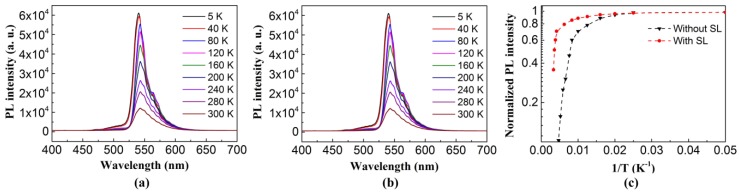
PL spectra of green LEDs (**a**) without and (**b**) with an InGaN/GaN SL; (**c**) Arrhenius plot of the normalized integrated PL intensity of the green MQWs without an SL and with an SL.

Figure 6a,b show the PL spectra of green LEDs without and with an InGaN/GaN SL. A S-shaped (decrease-increase-decrease) temperature dependence of the peak energy with increasing temperature from 5 K to 300 K was observed in Figure 6a,b, which was attributed to the inhomogeneity and carrier localization in the InGaN/GaN MQW [40]. The peak wavelength shifts of the green LED without and with an InGaN/GaN SL were 3.6 and 2.5 nm, respectively, when the temperature was increased from 5 K to 300 K. The smaller peak wavelength shift of the green LED with an InGaN/GaN SL was attributed to the reduced strain in the MQWs due to the insertion of an InGaN/GaN SL. The temperature dependence of the normalized integrated PL intensity, as shown in Figure 6c, can be described by the empirical Arrhenius equation [41]

(1) $$I\left(T\right)=\frac{I_{0}}{1\;+\;Cexp\left(-\frac{E_{a}}{k_{B}T}\right)},$$

where *I*_0_ is the PL intensity at low temperature, *C* is a constant proportional to the density of non-radiative recombination centers, *E_a_* is the activation energy of non-radiative recombination centers, *T* is the temperature, and *k_B_* is the Boltzmann constant. The fitting curves give the activation energy *E_a_* of 15.5 meV for the green MQW without an InGaN/GaN SL and 16.4 meV for the green MQW with an InGaN/GaN SL. The higher activation energy *E_a_* of non-radiative recombination centers is indicative of the superior light emission efficiency of the green MQW with an InGaN/GaN SL. The constant *C* is 2.4 and 0.8 for the green MQWs without and with an InGaN/GaN SL, respectively, indicating that there was a lower density of non-radiative recombination centers in the green MQW with an InGaN/GaN SL. 

By assuming that the non-radiative channels are suppressed at a low temperature, the ratio of the spectrally integrated PL intensity at room temperature (300 K) to that at a low temperature can be used to estimate the internal quantum efficiency (IQE) of green LEDs [42,43]. The IQE of green LEDs is given by the following equation

(2) $$IQE=\frac{I_{300K}}{I_{0K}}.$$

In a realistic measurement, *I*_0*K*_ could be replaced by *I*_5*K*_. Here, the *IQE* of green LEDs without and with an InGaN/GaN SL were estimated to be 20.07% and 29.35%, respectively. The improvement in *IQE* of the green LED with an InGaN/GaN SL was attributed to the large V-pits having a higher potential barrier height, which could effectively suppress the lateral diffusion of the carriers into TDs and thus result in a reduction in the non-radiative combination rate.

**Figure 7:**
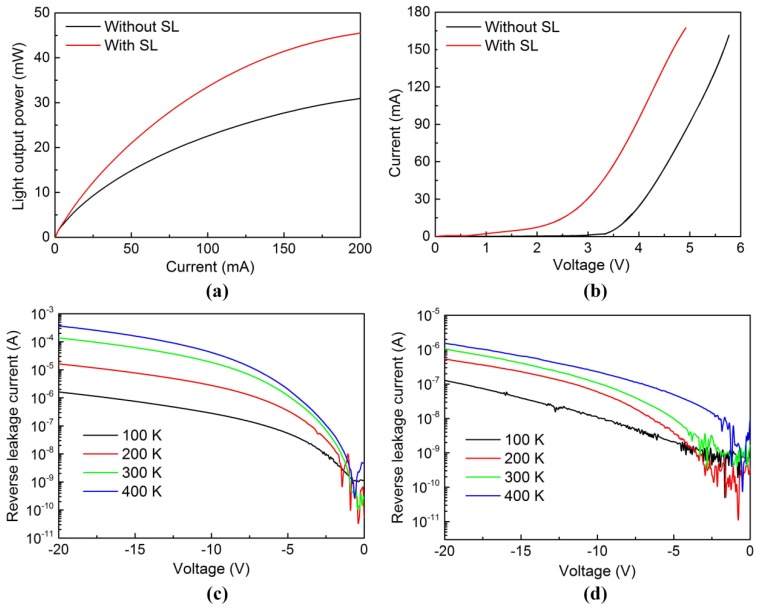
(**a**) Light output power versus current and (**b**) current versus voltage characteristics of green LEDs without and with an InGaN/GaN SL. Temperature-dependent current-voltage characteristics of green LEDs (**c**) without and (**d**) with an InGaN/GaN SL under the reverse bias condition.

Figure 7a shows the light output power as a function of injection current for green LEDs. At 20 mA, the light output power of the green LEDs without and with an InGaN/GaN SL was 8.2 and 10.6 mW, respectively. The external quantum efficiency (EQE) of green LEDs is described by

(3) $$EQE=\frac{P/hv}{I/e}=\frac{P\lambda }{I}\times \frac{e}{hc}=\frac{P\lambda }{1240\times I}$$

where *P* is the light output power of green LEDs, *I* is the injection current, and λ is the light emission wavelength. The light emission wavelength of the grown green LEDs was 542 nm. The corresponding EQEs were estimated to be 17.9% and 23.2% for the 542 nm green LEDs without and with an InGaN/GaN SL, respectively, at 20 mA. The *EQE* of the green LED with an InGaN/GaN SL was 29.6% higher than that of the green LED without an InGaN/GaN SL. The result revealed that larger V-pits having a higher potential barrier height can more effectively suppress the non-radiative recombination of the carrier at TDs, thereby leading to an improvement in the *EQE* of a green LED. Figure 7b shows the current-voltage characteristic curves of green LEDs. At 20 mA, the forward voltages of the green LEDs without and with an InGaN/GaN SL were 3.93 and 2.71 V, respectively. Owing to the lower polarization charge densities at the InGaN/GaN interfaces from the V-pit sidewalls, the effective barrier height for holes injected from the V-pit sidewalls is lower than that for holes injected from the flat MQW [44,45,46]. As a result, the injection of holes into the MQW via the sidewalls of the V-pits is easier than via the flat region. On the other hand, the surface coverage ratio of V-pits in the InGaN/GaN MQW was calculated to be about 0.02 and 0.12 for green LED without and with an InGaN/GaN SL, respectively. The higher surface coverage ratio of V-pits was favorable for the injection of holes into MQWs from the sidewall of the V-pits. Consequently, the green LED with an InGaN/GaN SL exhibits a lower forward voltage than that of the green LED without an InGaN/GaN SL.

Figure 6d and Figure 7c show temperature-dependent current-voltage characteristics of green LEDs under the reverse bias condition for temperatures ranging from 100 K to 400 K. We found that a higher reverse voltage tended to enhance the reverse leakage current for both of the green LEDs without and with an InGaN/GaN SL, which was attributed to the thermal activation of carriers from deep centers enhanced by an electric field. However, the reverse leakage current of the green LED with an InGaN/GaN SL was much lower than that of the green LED without an InGaN/GaN SL under the same reverse bias. For example, the leakage current of green LEDs without and with an InGaN/GaN SL were 18.8 and 0.109 μA, respectively, at −10 V and 300 K. It has been reported in our previous work that hopping conduction, including variable-range hopping (VRH) conduction and nearest-neighbor hopping (NNH) conduction, which is defined as carrier transport via electron hopping among localized states within the bandgap, is believed to the main mechanism causing the reverse leakage current [47]. The localized states are believed to be associated with TDs. Therefore, screening of TDs by the V-pits is considered to be an effective method for reducing the reverse leakage current. Additionally, the larger V-pits were also more effective in reducing the reverse leakage current of LEDs due to their higher Poole–Frenkel barrier height [24,48,49]. As a result, the reverse leakage current of the green LEDs decreased with increasing V-pit diameter.

## 4. Conclusions

We systematically investigated the effect of a V-pits-embedded InGaN/GaN SL on the optical and electrical properties of green LEDs. By performing a CL measurement, we have demonstrated that a potential barrier formed by a V-pit occurs around the TDs, and that the V-pit potential barrier height rises as the size of the V-pit increases, suggesting that a larger V-pit could more effectively hinder the non-radiative recombination of carrier at TDs. As a result, the EQE of green LEDs is improved by 29.6% at an injection current of 20 mA after implementing the V-pits-embedded InGaN/GaN SL. We observed that the forward voltage of green LEDs decreased with increasing V-pit diameter due to the enhanced holes injection. In addition, we found that the reverse leakage current of green LEDs decreased with increasing V-pit diameter, which was attributed to a more effective screening of TDs and also to a higher Poole–Frenkel barrier height.
